# The anti-carcinogenesis properties of erianin in the modulation of oxidative stress-mediated apoptosis and immune response in liver cancer

**DOI:** 10.18632/aging.102456

**Published:** 2019-11-20

**Authors:** Xinrui Zhang, Yingwu Wang, Xin Li, Anhui Yang, Zhiwen Li, Di Wang

**Affiliations:** 1 School of Life Sciences, Jilin University, Changchun 130012, China; 2 Zhuhai College of Jilin University, Jilin University, Zhuhai 519041, China; 3 Department of Anesthesiology, The First Hospital of Jilin University, Changchun 130021, China

**Keywords:** erianin, liver cancer, oxidative stress, immune response, mitochondria

## Abstract

In this study, erianin was found to reduce the viability of cancer cells, inhibit their proliferation and migration, induce G2/M phase arrest, enhance cancer cell apoptosis, promote an increase in levels of intracellular reactive oxygen species and a decrease in mitochondrial membrane potential, and regulate the expression levels of anti- and pro-apoptosis-related proteins in HepG2 and SMMC-7721 cells. Erianin inhibited tumor growth in HepG2- and SMMC-7721-xenograft tumor nude mouse models, reduced the expression levels of anti-apoptosis proteins and enhanced the expression levels of pro-apoptosis proteins in tumor tissues. Erianin inhibited tumor growth in immunosuppressed BALB/c mice bearing heterotopic tumors. Among 111 types of cytokines detected in proteome profiling of tumor tissues, erianin substantially influenced levels of 38 types of cytokines in HepG2-xenografted tumors and of 15 types of cytokines in SMMC-7721-xenografted tumors, most of which are related to immune functions. Erianin strongly affected the serum levels of cytokines, and regulated the activation of nuclear factor-kappa B (NF-κB), and the expression levels of nuclear factor erythroid 2-related factor 2 (Nrf2) and its downstream proteins in spleen. The anti-liver cancer properties of erianin were found to be related mostly to its modulation of oxidative stress-mediated mitochondrial apoptosis and immune response.

## INTRODUCTION

Liver cancer is a common malignant tumor, ranking third as the cause of cancer-related deaths worldwide [1]. In China, liver cancer kills nearly 383,000 patients each year [2]. Despite some breakthroughs in the early treatment of liver cancer, unfortunately, 50% of liver cancer cases are diagnosed at a late stage [3].

As the body’s chief detoxification organ, the liver is sensitive to oxidative stress, which is responsible for cell apoptosis, a prominent feature of liver disease [4]. Oxidative stress leads to the over-accumulation of intracellular reactive oxygen species (ROS) and/or dysfunction of mitochondrial metabolism, leading to mitochondrial apoptosis [5]. Nuclear factor erythroid 2-related factor 2 (Nrf2) is an important factor controlling oxidative stress and can regulate the expressions of antioxidant enzymes such as heme oxygenase-1 (HO-1) [6]. The HO-system is considered to be central to the stress response [7] and to protect the mitochondrial membrane potential via the inhibition of oxidative damage [8].

Accumulating research studies suggest that, rather than occurring independently, carcinogenesis is associated with chronic inflammation and immune dysfunction [9, 10]. Inflammation can induce chromosomal instability, enhance tumor cell proliferation and resistance to apoptosis, and stimulates angiogenesis and tissue remodeling [11–13]. Oxidative stress can activate nuclear factor-kappa B (NF-κB), exacerbating inflammation by regulating cytokines such as matrix metalloproteinases (MMPs) and interleukins [14–16]. The activation of NF-κB depends on the phosphorylation and ubiquitination of NF-κB inhibitor alpha (IKB-α), a process regulated by IKB kinase (IKK). Stimulation of NF-κB by extracellular regulated protein kinase (Erk) signals helps to regulate cell proliferation and survival [17].

Current treatments for liver cancer such as chemotherapy inevitably cause damage to the body's immune system [18]. Biological immunotherapy of liver cancer has become an important research topic, and traditional Chinese medicine is an important feature of liver cancer treatment [19]. Dendrobium candidum has been reported to have immune-enhancing and anti-inflammatory properties [20]. Erianin, a natural compound derived from Dendrobium candidum, shows various pharmacological activities [21]. The antioxidant activity of erianin was first reported by Dr. Tzi Bun Ng [22], and its inhibitory effects on the growth of cancer cells such as leukemia HL-60 cells, human hepatoma Bel7402 cells, and melanoma A375 cells, were subsequently elucidated [23, 24]. Erianin can effectively induce apoptosis, cause G2/M phase arrest, and inhibit xenografted tumor growth of osteosarcoma cells through regulation of oxidative stress [25]. Another study showed that erianin inhibits the proliferation and migration of breast cancer T47D cells and induces apoptosis of those cells by down-regulating B-cell lymphoma-2 (Bcl-2) expression and activating the caspase pathway [26]. However, few studies have examined erianin to reveal its anti-carcinogenic properties and the underlying mechanisms in the treatment of liver cancer.

This study investigated the anti–liver cancer properties of erianin in HepG2 and SMMC-7721 cells and in xenografted tumor nude mice and BALB/c mice models. In *in vitro* experiments, erianin induced cell apoptosis via its alterations of mitochondrial function. In *in vivo* experiments, erianin inhibited the growth of HepG2 and SMMC-7721-xenografted tumors in both nude mice and BALB/c mice via the regulation of oxidative stress and inflammatory conditions. The cumulative data provide the experimental evidence for the potential clinical value of erianin in therapy of liver cancer.

## RESULTS

### Erianin induced apoptosis in liver cancer cells via the regulation of mitochondrial function

**Figure 1:**
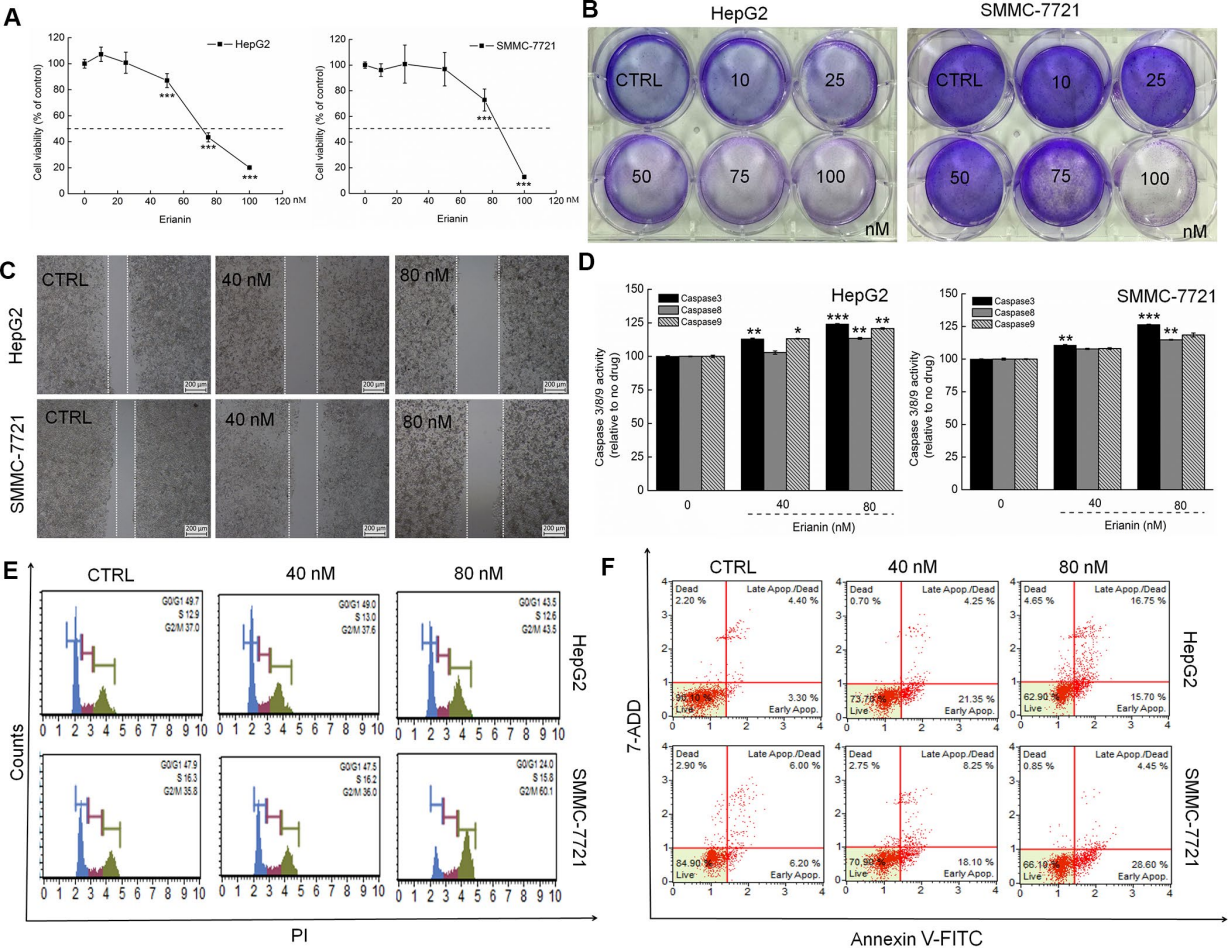
**Erianin showed toxicity toward liver cancer cells.** (**A**) Erianin reduced HepG2 and SMMC-7721 cell viability in a dose-dependent manner after a 24-h treatment. (**B**) Erianin significantly inhibited the formation of HepG2 and SMMC-7721 cell colonies (crystal violet staining, *n* = 6). (**C**) Erianin inhibited HepG2 and SMMC-7721 cell migration (migration assay, *n* = 6; 4× magnification, scale bar: 200 μm). (**D**) Erianin enhanced caspase-3, -8, and -9 activation in HepG2 and SMMC-7721 cells. Data are expressed as percentages relative to the corresponding control cells and as mean ± SD (*n* = 6). *P < 0.05, **P < 0.01, and ***P < 0.001 vs control cells. (**E**) Erianin increased the G2/M phase proportion within the cell cycle distribution (*n* = 6). (**F**) Erianin induced liver cancer cell apoptosis (*n* = 6).

The IC50 values of erianin on HepG2 and SMMC-7721 cells for a 24-h exposure were 43.69 nM and 81.02 nM, respectively (*P* < 0.001, Figure 1A). Erianin significantly inhibited the formation of HepG2 and SMMC-7721 cell colonies (*P* < 0.05, Figure 1B and Supplementary Figure 1A). In the cell scratch test, the migration ability of liver cancer cells was significantly inhibited after 24 h of co-culture with erianin (*P* < 0.05, Figure 1C and Supplementary Figure 1B). After a 24-h exposure to erianin, the activities of caspase-3, -8, and -9 (serving as apoptosis markers) were strongly enhanced (*P* < 0.001, Figure 1D). Compared with non-treated cells, erianin at 80 nM caused significant G2/M phase arrest in HepG2 and SMMC-7721 cells (Figure 1E). Erianin, especially at 80 nM, caused 32.45% and 33.05% of early/late apoptosis in HepG2 and SMMC-7721 cells, respectively (Figure 1F).

**Figure 2:**
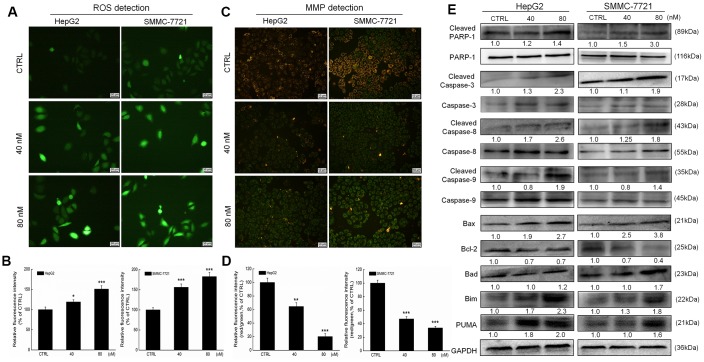
**Erianin induced mitochondrial apoptosis in liver cancer cells.** Erianin (**A**) increased intracellular reactive oxygen species (ROS) production and (**C**) decreased the mitochondrial membrane potential (20× magnification, scale bar: 50 μm). Qualitative data are expressed as (**B**) the green fluorescence intensity and (**D**) the ratio of red to green fluorescence intensity. Data are expressed as percentages relative to the corresponding control cells and mean ± SD (*n* = 6). *P < 0.05, **P < 0.01 and ***P < 0.001 vs control cells. (**E**) Erianin significantly enhanced the ratio of cleaved PARP/PARP, cleaved caspase-3/caspase-3, cleaved caspase-8/caspase-8 and cleaved caspase-9/ caspase-9, and the expression levels of Bax, Bad, Bim and PUMA, and reduced the expression levels of Bcl-2 in HepG2 and SMMC-7721 cells. Quantitative protein expression data were normalized to GAPDH expression levels in the corresponding samples. The marked average changes of proteins were expressed as folds relative to the corresponding control cells (*n* = 6).

The over-accumulation of intracellular ROS activates the mitochondrial apoptotic pathway [1]. Erianin increased the intracellular ROS levels in both HepG2 and SMMC-7721 cells, as indicated by the enhanced green fluorescence (*P* < 0.05, Figure 2A, 2B). A 6-h erianin exposure resulted in significant dissipation of MMP in liver cancer cells, as suggested by the reduced red fluorescence intensity and enhanced green fluorescence intensity (*P* < 0.01; Figure 2C, 2D).

Poly (ADP-ribose) polymerase (PARP) can be cleaved during cell apoptosis by caspases [27]. Erianin, especially at 80 nM, increased the expression levels of cleaved PARP, cleaved caspase-3, -8, and -9, and their ratio compared with related total protein levels in HepG2 and SMMC-7721 cells (Figure 2E).

Bcl-2 family members serve as important indices of mitochondrial function [1]. Incubation with erianin at 40 and 80 nM for 24 h strongly reduced the expression levels of Bcl-2 and enhanced the expression levels of Bax, Bad, Bim and p53 upregulated modulator of apoptosis (PUMA) in HepG2 and SMMC-7721 cells compared with the control cells (Figure 2E).

### Erianin inhibited HepG2- and SMMC-7721-xenografted tumor growth in nude mice as a result of its pro-apoptotic property

**Figure 3:**
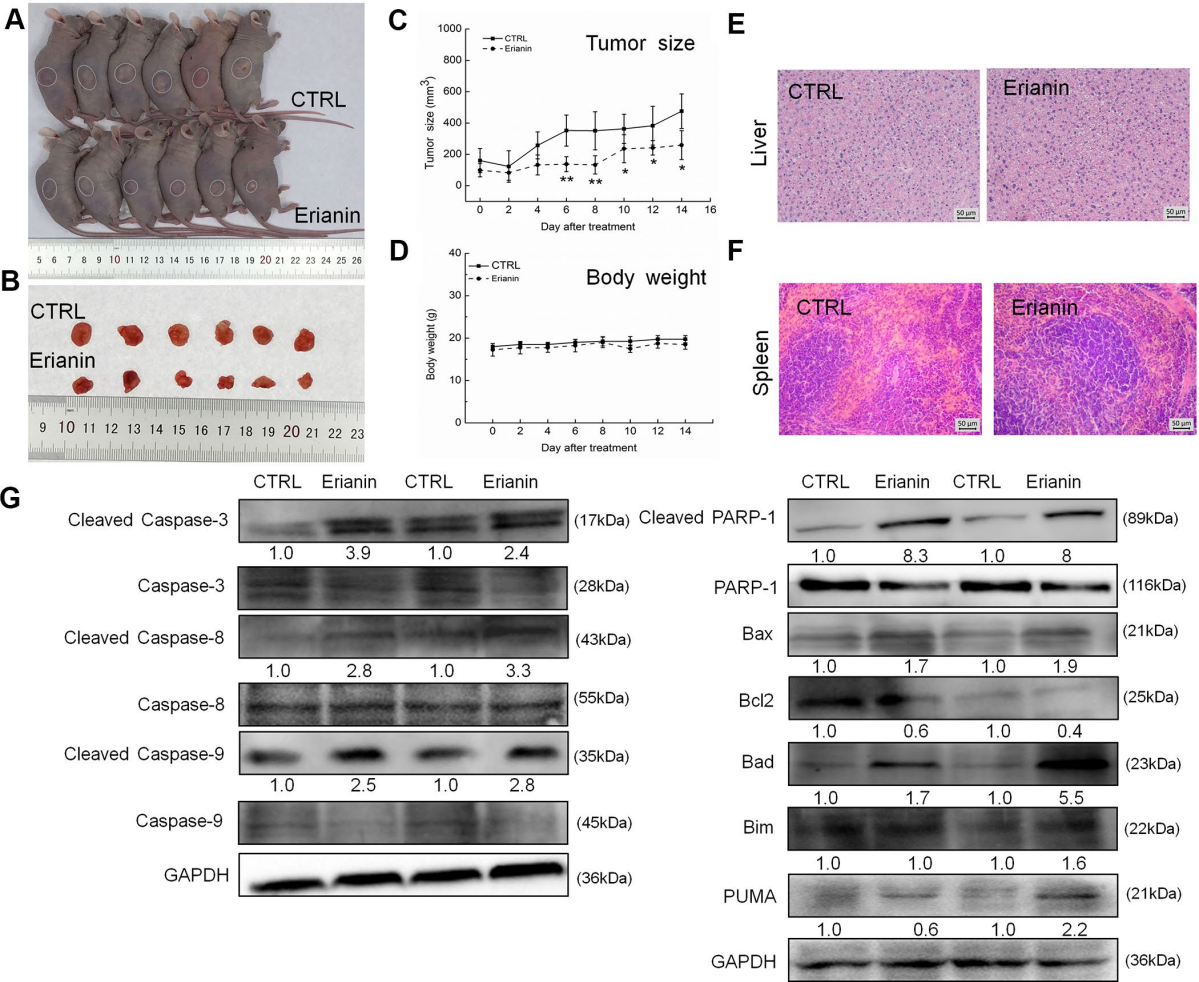
**Erianin inhibited HepG2-xenograft tumor growth in BALB/c nude mice.** BALB/c athymic nude mice inoculated with HepG2 cells were treated with erianin (20 mg/kg dissolved in 0.9% saline solution containing 1:10,000 DMSO) or vehicle solvent (0.9% saline solution containing 1:10,000 DMSO) for 14 days. (**A**) Tumor-bearing nude mice and (**B**) tumors collected from vehicle and erianin-treated groups. (**C**) Tumor volumes were measured every other day. Tumor sizes are expressed as mean ± SD (*n *= 6). * *P *< 0.05, ** *P *< 0.01 vs control group. (**D**) Mean (±SD) body weights in the erianin-treated and vehicle groups (*n *= 6). Pathological analysis of (**E**) liver and (**F**) spleen tissues via H&E staining. (**G**) Erianin significantly enhanced the ratio of cleaved PARP/PARP, cleaved caspase-3/caspase-3, cleaved caspase-8/caspase-8 and cleaved caspase-9/caspase-9, and the expression levels of Bax, Bad, Bim and PUMA, and reduced the expression levels of Bcl-2 in tumor tissues. Quantitative protein expression data were normalized to GAPDH levels and/or related total protein levels in the corresponding samples. The marked average changes of proteins were expressed as folds relative to the corresponding control tumor tissues (*n *= 6).

**Figure 4:**
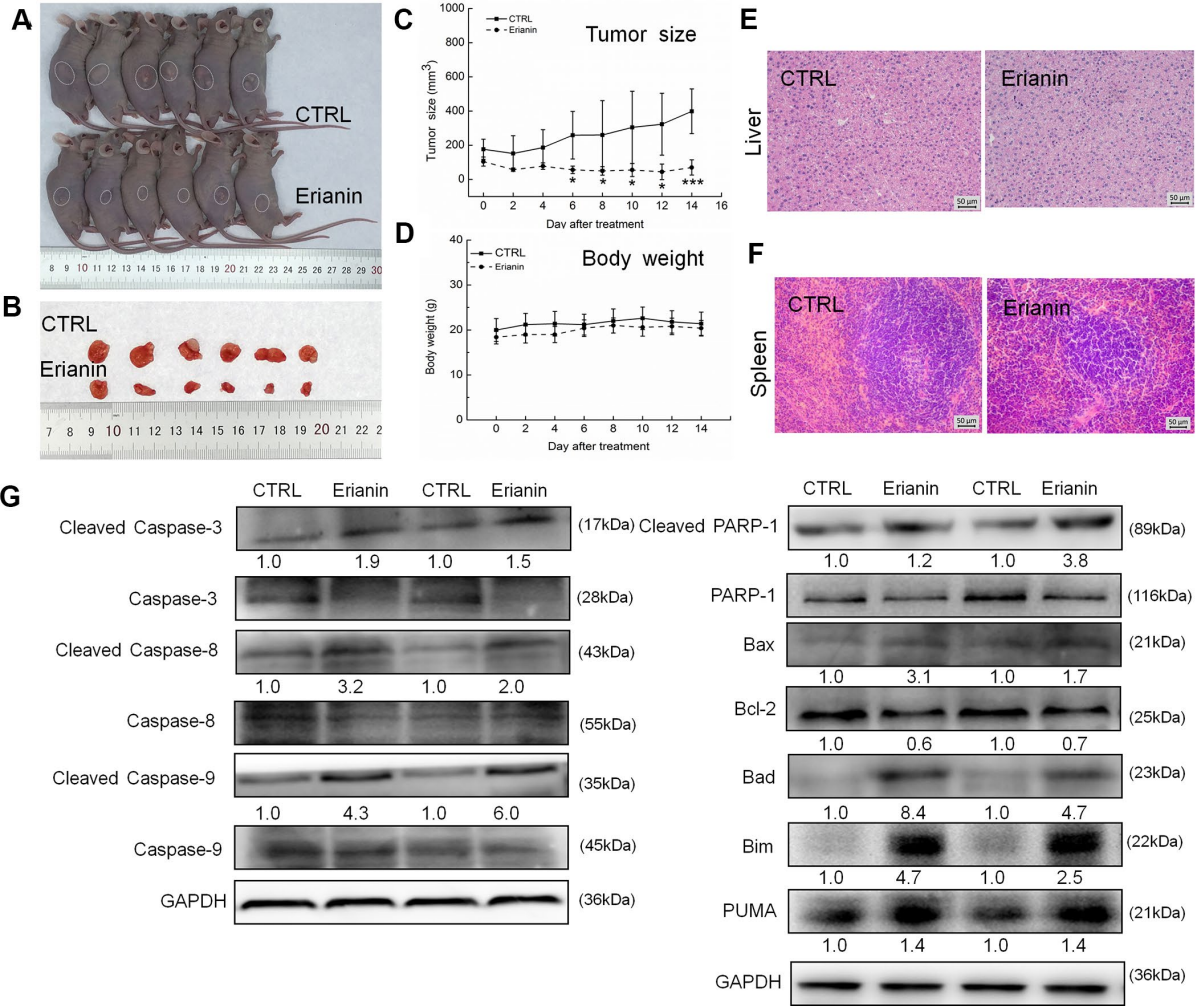
**Erianin inhibited SMMC-7721-xenograft tumor growth in BALB/c nude mice.** BALB/c athymic nude mice inoculated with SMMC-7721 cells were treated with erianin (20 mg/kg dissolved in 0.9% saline solution containing 1:10,000 DMSO) or vehicle solvent (0.9% saline solution containing 1:10,000 DMSO) for 14 days. (**A**) Tumor-bearing nude mice and (**B**) tumors collected from vehicle and erianin-treated groups. (**C**) Tumor volumes were measured every other day. Tumor sizes are expressed as mean ± SD (*n* = 6). * *P* < 0.05, ** *P* < 0.01 and *** *P* < 0.001 vs control group. (**D**) Mean (±SD) body weights in the erianin-treated and vehicle groups (*n* = 6). Pathological analysis of (**E**) liver and (**F**) spleen tissues via H&E staining. (**G**) Erianin significantly enhanced the ratio of cleaved PARP/PARP, cleaved caspase-3/caspase-3, cleaved caspase-8/caspase-8 and cleaved caspase-9/caspase-9, and the expression levels of Bax, Bad, Bim and PUMA, and reduced the expression levels of Bcl-2 in tumor tissues. Quantitative protein expression data were normalized to GAPDH levels and/or related total protein levels in the corresponding samples. The marked average changes of proteins were expressed as folds relative to the corresponding control tumor tissues (*n* = 6).

The tumor-xenografted nude mice were used to investigate the pro-apoptotic effects of erianin. The 14-day erianin treatment at 20 mg/kg strongly inhibited the growth of HepG2-xenografted tumors (*P* < 0.05; 258.9 mm^3^ (erianin-treated mice) *vs.* 475.8 mm^3^ (control mice), Figure 3A–3C) and that of SMMC-7721-xenografted tumors (*P* < 0.05; 70.2 mm^3^ (erianin-treated mice) *vs.* 398.8 mm^3^ (control mice), Figure 4A–4C) without influencing their body weights (Figures 3D, 4D). Hematoxylin and eosin (H&E) staining showed that the liver cells and spleen cells of CTRL and erianin groups were uniform in structure and had no inflammatory infiltration, indicating that erianin did not change the organ structure (Figures 3E, 3F, 4E, 4F).

Compared with non-treated nude mice, 14-day erianin treatment resulted in significant increments in the ratio of cleaved PARP/PARP, cleaved caspase-3, -8, and -9/caspase-3, -8 and -9, and the expression levels of Bax, Bad, Bim and PUMA, and reduction in the expression levels of Bcl-2 in HepG2-xenografted tumors (Figure 3G) and SMMC-7721-xenografted tumors (Figure 4G).

### Erianin inhibited HepG2- and SMMC-7721-xenografted tumor growth in BALB/c mice via regulation of immune function

**Figure 5:**
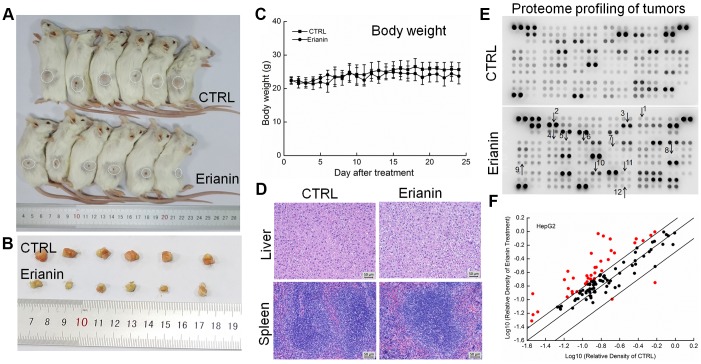
**Erianin inhibited HepG2-xenograft tumor growth in BALB/c mice.** After three consecutive injections of cyclophosphamide, BALB/c mice inoculated with HepG2 cells were treated with erianin (20 mg/kg dissolved in 0.9% saline solution containing 1:10,000 DMSO) or vehicle solvent (0.9% saline solution containing 1:10,000 DMSO) for 24 days. (**A**) Tumor-bearing mice and (**B**) tumors collected from vehicle- and erianin-treated groups. (**C**) Mean (±SD) body weights in the erianin-treated and vehicle groups (*n* = 6). (**D**) Pathological analysis of liver and spleen tissues via H&E staining. (**E**) Effects of erianin on 111 types of cytokines in mice tumors detected by Mouse XL Cytokine Kit. The arrows indicate the factors for further detection. 1. CCL2; 2. CCL11; 3. CCL21; 4. CXCL11; 5. CXCL13; 6. CXCL16; 7. GM-CSF; 8. IL-6; 9. IL-10; 10. MMP-2; 11. MMP-9; 12. TNF-α. (**F**) Scatter diagram of 111 cytokines. The relative density is the ratio of the absolute value and the reference spot value. The red dots indicate the factors with a change of >50% compared with control mice.

**Figure 6:**
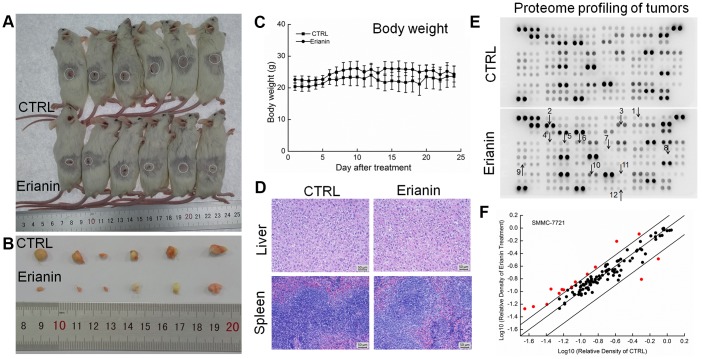
**Erianin inhibited SMMC-7721-xenograft tumor growth in BALB/c mice.** After three consecutive injections of cyclophosphamide, BALB/c mice inoculated with SMMC-7721 cells were treated with erianin (20 mg/kg dissolved in 0.9% saline solution containing 1:10,000 DMSO) or vehicle solvent (0.9% saline solution containing 1:10,000 DMSO) for 24 days. (**A**) Tumor-bearing mice and (**B**) tumors collected from vehicle- and erianin-treated groups. (**C**) Mean (±SD) body weights in the erianin-treated and vehicle groups (*n* = 6). (**D**) Pathological analysis of liver and spleen tissues via H&E staining. (**E**) Effects of erianin on 111 types of cytokines in mice tumors detected by Mouse XL Cytokine Kit. The arrows indicate the factors for further detection. 1. CCL2; 2. CCL11; 3. CCL21; 4. CXCL11; 5. CXCL13; 6. CXCL16; 7. GM-CSF; 8. IL-6; 9. IL-10; 10. MMP-2; 11. MMP-9; 12. TNF-α. (**F**) Scatter diagram of 111 cytokines. The relative density is the ratio of the absolute value and the reference spot value. The red dots indicate the factors with a change of >50% compared with control mice.

The tumor-xenografted BALB/c mice were used to further investigate the inhibitory effects of erianin on tumor growth. A 24-day erianin treatment at 20 mg/kg strongly inhibited the growth of HepG2-xenografted tumors (Figure 5A, 5B) and SMMC-7721-xenografted tumors (Figure 6A, 6B) without influencing the animals’ body weight (Figures 5C, 6C). According to H&E staining, in both CTRL and erianin-treated mice, the cells in liver and spleen were uniform in structure, and no inflammatory infiltration were noted in the organs (Figures 5D, 6D).

To systematically investigate the inhibitory effects of erianin on tumor growth and the possible mechanisms, a Mouse XL Cytokine Kit containing 111 types of cytokines was used to detect changes in cytokines levels in tumor tissues. Compared to the control mice, erianin at 20 mg/kg substantially influenced the levels of 38 types of cytokines in HepG2-xenografted tumors (Figure 5E and 5F, Supplementary Table 1), and of 15 types of cytokines in SMMC-7721-xenografted tumors (Figure 6E and 6F, Supplementary Table 1), most of which are associated with immune functions.

**Figure 7:**
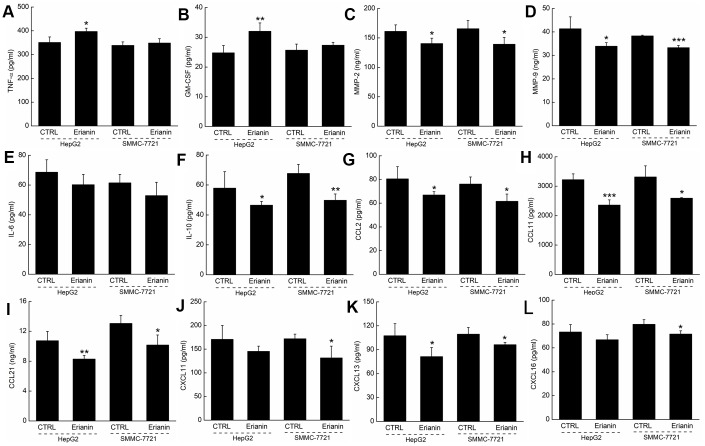
**Effects of erianin on the immune factors of serum in tumor- xenografted mice.** Compared with control mice, erianin enhanced the serum levels of (**A**) TNF-α and (**B**) GM-CSF, and reduced the serum levels of (**C**) MMP-2, (**D**) MMP-9, (**F**) IL-10, (**G**) CCL2, (**H**) CCL11, (**I**) CCL21, (**J**) CXCL11, (**K**) CXCL13 and (**L**) CXCL16, but failed to influence the levels of (**E**) IL-6. Data are represented as means ±SD (*n* = 6), relative to the control group: *P < 0.05, **P < 0.01 and ***P < 0.001.

Based on the results obtained from proteome profiling, 12 types of immunological correlation factors in the serum of BALB/c were analyzed via enzyme-linked immunosorbent assay (ELISA). In the mice bearing HepG2- and SMMC-7721-xenografted tumors, erianin enhanced the serum levels of tumor necrosis factor-α (TNF-α, contributing to immune response and cell apoptosis) (*P* < 0.05) (Figure 7A) and granulocyte-macrophage colony stimulating factor (GM-CSF, enhancing the activation of T cells and the killing function of T cells on tumor cells) (*P* < 0.01) (Figure 7B), and reduced the serum levels of matrix metalloproteinase 2 (MMP-2, promoting the invasion, metastasis, and angiogenesis of tumors [28]) (*P* < 0.05) (Figure 7C), matrix metalloproteinase 9 (MMP-9, serving as a biomarker for cancer detection [29]) (*P* < 0.05) (Figure 7D), IL-10 (known as an inhibitor of inflammation) (*P* < 0.05) (Figure 7F), CCL2 (MCP-1, product by macrophages and indirectly promoting the invasion and metastasis of tumors [30]) (*P* < 0.05) (Figure 7G), CCL11 (promoting blood vessel growth of tumors [31]) (*P* < 0.05) (Figure 7H), CCL21 (mediating the metastasis of tumor cells to lymph nodes [32]) (*P* < 0.05) (Figure 7I), CXCL11 (promoting tumor progression [33]) (*P* < 0.05) (Figure 7J), CXCL13 (responsible for the imbalances in microenvironments of B cells [34]) (*P* < 0.05) (Figure 7K) and CXCL16 (markers of cancer cell production in inflammatory environment [35]) (*P* < 0.05) (Figure 7L).

### The regulation of Nrf2 and NF-κB signaling is involved in erianin-mediated tumor growth inhibition

**Figure 8:**
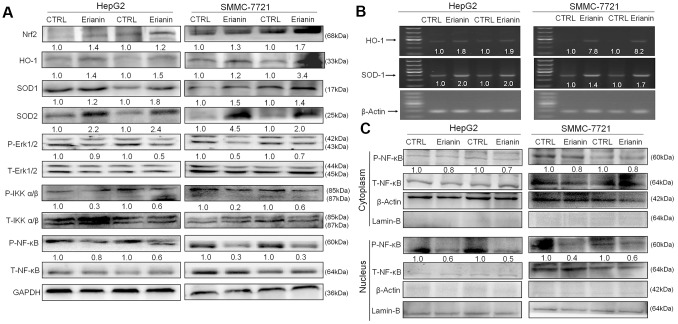
**Effects of erianin on oxidative stress-mediated NF-κB pathway.** (**A**) Erianin enhanced the levels of Nrf2, HO-1, SOD-1, and SOD-2, and reduced the phosphorylation levels of Erk1/2, IKKα/β, and NF-κB in spleens of BALB/c mice bearing HepG2- and SMMC-7721-xenografted tumors. Quantitative protein expression was normalized to GAPDH levels and/or related total protein levels in the corresponding samples. The marked average changes of proteins were expressed as folds relative to the corresponding control tumor tissues (*n* = 6). (**B**) Erianin enhanced the RNA levels of HO-1 and SOD-1 in spleens of BALB/c mice bearing HepG2- and SMMC-7721-xenografted tumors. Quantitative RNA expression data were normalized to the corresponding β-actin levels. The marked average changes of HO-1 and SOD-1 were expressed as folds relative to the corresponding control tumor tissues (*n* = 6). The molecular mass from top to bottom of the marker is: 1000, 700, 500, 400, 300, 200, 100 (bp). (**C**) Erianin reduced the phosphorylation levels of NF-κB in cytoplasm, and inhibited its transfer from cytoplasm to nucleus. The quantitative P-NF-κB expression in the cytoplasm and nucleus was normalized to the T- NF-κB levels, respectively. The marked average changes were expressed as folds relative to the corresponding control tumor tissues (*n* = 6).

We infer that the tumor-inhibitory properties of erianin are related to oxidative stress-mediated immune function. The activation of Nrf2 can increase the expression of antioxidant proteins, helping to decrease oxidative stress, regulating cell proliferation, suppressing inflammation, and influencing the immune responses [36, 37]. The 24-day erianin administration enhanced the expression levels of Nrf2, HO-1, superoxide dismutase-1 (SOD-1), and superoxide dismutase-2 (SOD-2) in spleens of HepG2- and SMMC-7721-xenografted tumors from BALB/c mice (Figure 8A). Furthermore, erianin reduced the phosphorylation levels of Erk1/2, IKKα/β, and NF-κB in spleens of HepG2- and SMMC-7721-xenografted tumors from BALB/c mice (Figure 8A). Via detection using reverse transcription-polymerase chain reaction (RT-PCR), the 24-day erianin administration enhanced the gene expression levels of HO-1 and SOD-1 in spleens of BALB/c mice bearing with HepG2- and SMMC-7721-xenografted tumors (Figure 8B). NF-κB can be activated under the oxidative environment, and then transfers to the nucleus [38]. In spleen of BALB/c mice bearing with HepG2- and SMMC-7721-xenografted tumors, erianin suppressed the transfer of P-NF-κB from cytoplasm to nucleus (Figure 8C).

## DISCUSSION

In this study, the anti-liver cancer effects of erianin were confirmed in HepG2 and SMMC-7721 cells and in xenografted tumor nude mice and BALB/c mice models. In *in vivo* experiments, erianin exerted little influence on body weight and organ (liver and spleen) structure, indicating its safety for use in the mice in our experiments.

During the pro-apoptotic process on liver cancer cells, erianin caused the over-accumulation of intracellular ROS and reduced the dissipation of MMP (considered as an index of mitochondrial apoptosis) [39, 40]. Cancer cells are more susceptible to intracellular ROS damage than are normal cells [41]. Overproduction of ROS induces mitochondrial permeability transition, interferes with mitochondrial transmembrane potential, and eventually causes oxidative damage to mitochondria, leading to further release of ROS from mitochondria to the cytoplasm [42]. In combination with the mitochondrial apoptosis, pro-apoptotic molecules, such as cytochrome c, released from mitochondria activate caspase-3 and -9, which cleave specific substrate proteins, such as PARP, to induce DNA damage [1]. In liver cancer cells and the tumor tissues collected from the nude mice, erianin enhanced the expression levels of Bax and Bad, and reduced the levels of Bcl-2 (which regulates MMP levels by formation of the Bcl-2/Bax heterodimer) [43]. BH3-only proteins, consisted in Bcl-2 family, act as a pro-apoptotic initiator, including PUMA and Bim, can directly activate Bax, triggering a pro-apoptotic process [44]. The high expression of Bax and Bad, the pro-apoptotic member of the Bcl-2 family, can disrupt ATP synthesis and help to activate the caspase signal transduction pathway [45]. Meanwhile, the imbalance in expression levels of pro- and anti-apoptotic members of the Bcl-2 family is responsible for the generation of ROS [46]; consequently, over-production of ROS activates caspase-8 and -9 [47]. In a feedback loop, the activated capase-8 not only directly activates caspase-3, but also links to the mitochondrial apoptosis through cleaved Bid [48]. The anti-liver cancer effects of erianin were confirmed in cells and nude mice, a possible mechanism might be the modulation of oxidative stress-mediated mitochondrial apoptosis.

Excessive inflammation is responsible for cancer development [49]. Compared with healthy volunteers, the extremely high serum levels of MMP-9 and CCL21 in patients with colon cancer [50], of CCL11 in patients with gastric cancer [31], of CCL2/MCP-1 in patients with breast cancer [51] and of CXCL13 in patients with liver cancer [34] have been reported. In immunosuppressed BALB/c mice bearing a hepatoma, erianin regulated the serum levels of MMPs, interleukins, and chemokines. MMPs, especially MMP-2 and MMP-9, participate in the angiogenesis of tumors and promote the invasion and metastasis of various tumors, which influence T cell proliferation [29, 52]. GM-CSF, a broad-spectrum glycoprotein, promotes the maturation of immature dendritic cells, up-regulates the expression of the major histocompatibility complex, and helps to enhance the killing effect of the immune system on tumors [53]. Chemokines disrupt the immune defense and promote tumor infiltration and metastasis via avoidance of uncontrolled killing, induction of tumor cell migration, stimulation of angiogenesis, and promotion of cell matrix degradation [54]. As reported, CXCL12/SDF-1 can enhance the invasive ability of the human pancreatic cancer cell PANC-1 by up-regulating the expression of vascular endothelial growth factor and MMP-9 [55]. The immune response related to cancer immunotherapy of GM-CSF, IL-6, and IL-10 notably involves modification of T-cell responses [56–58]. Encouragingly, many studies correlating with the effects of CCL2, CCL11, and CCL21 on anti-cancer properties focus on their relationships with T cell functions [59–61].

The potential of erianin to inhibit the growth of liver cancer tumors associated with its modulation of immune response was confirmed by detecting the protein expressions in spleen tissues of BALB/c mice bearing hepatoma. Under the normal conditions, NF-κB binds to the inhibitor IκBα with the inactivated form in the cytoplasm; however, under the tumor environment, the degradation of IκBα leads to the transfer of NF-κB into the nucleus [62]. NF-κB involved in the regulation of inflammatory factors including interleukins and MMP-9, is activated in most cancers via Erk1/2 and IKKα/β [63–65]. The activated Erk1/2 signaling not only regulates the expression of chemokines through NF-κB, but also directly participates in CCL21-mediated epithelial-mesenchymal transformation of lung cancer [66]. Erianin could suppress the activity of NF-κB via inhibiting the phosphorylation of Erk1/2 and IKKα/β, thus inhibiting the secretion of inflammatory factors.

Oxidative stress is not only responsible for apoptosis, but is also associated with immune response. The redox imbalance, caused by over-accumulation of ROS, is responsible for the transfer of Nrf2 from cytoplasm to nucleus, where it controls the expression of its downstream proteins, such as SOD-1/-2 and HO-1 [36]. This process is involved in cancer cell apoptosis [67], which has been also reported in our separate experiments [68]. Taxifolin curbs the carcinogenesis of 1,2-dimethyl hydrazine induced colon cells, leading to their apoptosis by activating the Nrf2 signaling pathway [69]. Meanwhile, Nrf2 can interfere with the production of interleukins [70], and can directly inhibit the expression of NF-κB [71]. Several agents show dual functions of anti-inflammatory and anti-oxidation via regulation of Nrf2/NF-κB signaling [72]. NF-κB is well known for its triggering immune escape of tumor cells via regulating inflammatory cytokines [73]. Moreover, NF-κB is abnormally activated in various malignant tumors. The suppression of NF-κB activation can trigger the extrinsic and intrinsic apoptosis in cancer cells [74]. The present data suggest that the anti-liver cancer property of erianin may be related to the modulation of oxidative stress-mediated apoptosis and immune response; however, their relationship linking by Nrf2 signaling still needs further investigation.

The current study still leaves some unanswered questions. Although the evidence obtained by detecting the cytokines suggests that erianin may influence the functions of T cells during its inhibition of tumor growth, we did not obtain direct results to confirm this event. In our on-going experiments, we have found that erianin can stimulate the proliferation of spleen T-lymphocytes in BABL/c mice with cyclophosphamide-induced immunosuppression. Additional experiments will be performed to confirm this possibility. Furthermore, in-depth research will explore the linkage of Nrf2 signaling to the apoptosis and the immune response.

## CONCLUSIONS

The anti-liver cancer effects of erianin were confirmed in liver cancer cells and their xenografted tumor-bearing mice via oxidative stress-mediated mitochondrial apoptosis and immune response, suggesting its potential for development as a drug therapy for liver cancer treatment.

## MATERIALS AND METHODS

### Cell culture

Liver cancer cells, HepG2 (CRL-11997) and SMMC-7721(BNCC33) obtained from the American Type Culture Collection (ATCC), were cultured in Dulbecco’s Modified Eagle Media (DMEM, Gibco) supplemented with 10% FBS (Zhejiang Tian hang bio Polytron Technologies Inc), 1% penicillin and streptomycin, and 0.1% plasmocin prophylactic (Gibco) at 37°C with 5% CO_2_.

### Cell viability assay

Cell viability was measured by the conversion of 3-(4,5-dimethylthiazolyl-2)-2,5-diphenyltetrazolium bromide (MTT, Source Leaf Biological Technology Co. Ltd., Shanghai, China) to formazan. Cells were seeded into 96-well plates at 6×10^4^ cells/mL. Cells were exposed to erianin (Source Leaf Biological Technology Co. Ltd., Shanghai, China; purity >98.0%) at doses of 10, 25, 50, 75, and 100 nM for 24 h. MTT (5 mg/mL) was then added and the cells incubated for 4 h at 37°C under darkness. Dimethyl sulfoxide (DMSO, Sinopharm Chemical Reagent Co, Ltd.) was used to dissolve the purple formazan crystals. The absorbance of each well was measured using the Synergy™4 Microplate Reader (BioTek Instruments, Winooski, VT, USA) at a wavelength of 490 nm.

### Colony formation assay

HepG2 and SMMC-7721 cells were seeded into 6-well plates at 5×10^4^ cells/well and exposed to erianin at doses of 0, 10, 25, 50, 75, and 100 nM for 7 days. After fixation and staining, the cells were washed and photographed. The crystal violet was dissolved with DMSO and the absorbance of each well was measured using the Synergy™4 Microplate Reader (BioTek Instruments, Winooski, VT, USA) at a wavelength of 590 nm.

### Migration assay

The cell migration ability was detected by cell scratch test. HepG2 and SMMC-7721 cells were plated into 6-well plates at 5×10^5^ cells/well. When cell cultures 90% confluence, the cells were scratched with a 10-μL micropipette tip and then exposed to erianin at doses of 40 and 80 nM for 24 h. Images were captured by an inverted microscope with an attached camera (Nikon Corp., Tokyo, Japan). The distances traveled by the migrating cells were quantified using the ImageJ software version 1.46 (National Institutes of Health, Bethesda, MD, USA) to evaluate the cell migratory ability.

### Assessment of caspase activities

Quantification of the relative caspase activities in erianin-treated cells was carried out using the respective assay kits (caspase-3, G015; caspase-8, G017; caspase-9, G018; Nanjing Jiancheng Bioengineering Institute, Nanjing, China). The liver cancer cells were plated into 6-well plates at 2×10^5^ cells/well and then exposed to erianin at 40 and 80 nM for 24 h. Treated cells were lysed, and the activities of caspase-3, -8, and -9 were evaluated according to the manufacturer’s protocols.

### Cell cycle and apoptosis analyses

The liver cancer cells were plated into 6-well plates at 2×10^5^ cells/well and exposed to erianin at 40 and 80 nM for 12 h. After fixation in 70% ethanol at 4°C overnight, cells were stained with Muse™ Cell Cycle reagent (Millipore, Billerica, MA, USA) under darkness at room temperature for 30 min. Cell cycle progression was analyzed using the Muse® Cell Analyzer (Millipore, Billerica, MA, USA).

The liver cancer cells were plated into 6-well plates at 2×10^5^ cells/well and exposed to erianin at 40 and 80 nM for 24 h. Cells were stained with Muse™ Annexin V and Dead Cell reagent (Millipore, Billerica, MA, USA) under darkness at room temperature for 20 min. Cell apoptosis was analyzed using the Muse® Cell Analyzer (Millipore, Billerica, MA, USA).

### Assessment of intracellular ROS levels and mitochondrial membrane potential (MMP)

HepG2 and SMMC-7721 cells were plated into 6-well plates at 2×10^5^ cells/well and incubated with erianin at 40 and 80 nM for 6 h. Treated cells were either incubated with fluorescent probe 2,7-dichlorofluorescein diacetate (DCFH-DA; Sigma-Aldrich, USA) (final concentration 10 μM) for 20 min at 37°C in darkness to analyze the intracellular levels of ROS, or stained with 5,5′,6,6′-Tetrachloro-1,1′,3,3′-tetraethylbenzimidazolylcarbocyanine iodide (JC-1; Sigma-Aldrich, USA) (final concentration 2 μM) for 15 min at 37°C in darkness to analyze the MMP changes. After washing, the fluorescence intensity was measured using an Eclipse TE 2000-S fluorescence microscope (Nikon Corp., Tokyo, Japan). The quantitative data were analyzed with the ImageJ software version 1.46.

### HepG2- and SMMC-7721-xenografted tumor mouse models

All animal experiments were conducted under the guidance of the Institutional Animal Care and Use Committee of Jilin University (NO. 2017SY0502).

### HepG2- and SMMC-7721-xenografted tumor model in BALB/c nude mice

Male BALB/c nude mice (5 weeks old) were purchased from Wei-tongli-hua Laboratory Animal Technology Company, Beijing, China. HepG2 and SMMC-7721 cells at a density of 8×10^6^ cells/100 μL were inoculated subcutaneously into the right dorsum (near the hind leg) of the nude mice. When the tumor volume reached ~100 mm^3^, all mice were randomly assigned to two groups (n = 6 each), and intraperitoneally injected with physiological saline containing DMSO (control mice) or 20 mg/kg of erianin (erianin-treated mice) every other day for seven injections. The tumors and body weight of the mice were measured individually before each administration. Tumor size was calculated as follows: length (mm) × (width (mm))^2^ × 0.5. After the last injection, mice were sacrificed, the tumors were removed and lysed for western blotting, and the liver and spleen tissues were fixed in formalin for histopathological examination.

### HepG2- and SMMC-7721-xenograft tumor model in BALB/c mice

Male BALB/c mice (8 weeks old) were purchased from Yis Laboratory Animal Technology Co., Ltd., Changchun, China. All mice received intraperitoneal injections of cyclophosphamide at 50 mg/kg once per day for 3 days. Fresh tumor samples obtained in HepG2 and SMMC-7721 heterotopic tumor model in nude mice were cleaned and sliced with a scalpel to 2 × 2 × 2 mm. The tumor masses were implanted at the right dorsum (near the hind leg) of the BALB/c mice. Tumor growth was observed daily. When the tumors reached a certain volume, the mice were randomly divided into two groups (n =6 each) and intraperitoneally injected with physiological saline containing DMSO (control mice) or erianin at 20 mg/kg (erianin-treated mice) once per day for 24 days. The peripheral blood from the orbital venous plexus of the mice was collected after the last therapy, and then all mice were sacrificed. Tumors, spleens and livers were collected and either stored at -80°C or fixed in formalin for further analysis.

### Histopathological examination

The livers and spleens collected from nude mice and BALB/c mice were fixed in formalin for 24 h and embedded in paraffin. Sections of 5 μm thickness were cut by microtome (Leica, Wetzlar, Germany) and stained with H&E. Images were captured from the Eclipse TE 2000-S fluorescence microscope (Nikon Corp., Tokyo, Japan).

### Proteome profiling of tumor tissues collected from BALB/c mice

The tumor tissues obtained from BALB/c mice were homogenized in radioimmunoprecipitation assay lysis buffer (Sigma-Aldrich, St. Louis, MO, USA) containing 1% protease inhibitor cocktail (Sigma-Aldrich, St. Louis, MO, USA). A Mouse XL Cytokine Kit (ARY028, R&D Systems, Millipore, USA) was used to analyze 111 cytokines contained in the tumor tissues lysate according to the manufacturer's protocols.

### Immune cytokines detection

The levels of immune-related cytokines in the serum of tumor-xenografted BALB/c mice, such as interleukin-6 (IL-6, CK-E20012), interleukin-10 (IL-10, CK-E20005), tumor necrosis factor-α (TNF-α, CK-E20220), granulocyte-macrophage colony stimulating factor (GM-CSF, 42921), matrix metalloproteinase 2 (MMP-2, CK-E20019), matrix metalloproteinase 9 (MMP-9, 42784), CCL2 (monocyte chemotactic protein 1, MCP-1, 42818), Eotaxin 1/CCL11 (CK-E20114), CCL21 (48385), ITAC/CXCL11 (40656), CXCL13 (48644), and CXCL16 (42751), were detected by commercial enzyme-linked immunosorbent assay (ELISA) kits purchased from the Source Leaf Biological Technology Co. Ltd., Shanghai, China.

### Western blot analysis

HepG2 and SMMC-7721 cells were plated into 6-well plates at 2×10^5^ cells/well and incubated with erianin at 40 and 80 nM for 24 h. Treated cells, tumor tissues obtained from BALB/c nude mice, and spleen tissues obtained from BALB/c mice were homogenized by radioimmunoprecipitation assay buffer containing 1% protease inhibitor cocktail and 2% PMSF (Sigma-Aldrich, St. Louis, MO, USA). The cytoplasm and nuclear protein in the spleens of BALB/c mice bearing with HepG2- and SMMC-7721-xenografted tumors were isolated using NE-PER Nuclear and Cytoplasmic Extraction Kit (Thermo Fisher Scientific, Waltham, MA, USA). The protein concentration of samples was analyzed by a Standard BCA Protein Assay Kit (Merck Millipore, Billerica, MA, USA) following the manufacturer's instructions. Forty-microgram samples were electrophoresed and separated with 10% SDS-PAGE gel at 90 V-120V, and then transferred onto PVDF membranes (0.45 μm, Merck Millipore, Billerica, MA, USA) at 100 V for 2 h. Membranes were blocked with 5% BSA (Genview, USA) in TBS and then incubated overnight at 4 °C with primary antibodies poly (ADP-ribose) polymerase (PARP-1, sc8007), cleaved PARP-1 (sc56196) (Santa Cruz, USA), superoxide dismutase-1 (SOD-1, bs-10216R), total (T)-caspase-3 (bs-0081R), Bim (bs-1488R), PUMA (bs-1573R), Lamin-B (bs-1840R) (Bioss Antibodies, China), cleaved caspase-3 (ab2302), T-caspase-8 (ab181580), T-caspase-9 (ab25758), Bad (ab32445), Bax (ab32503), B-cell lymphoma-2 (Bcl-2) (bsm-33047M), Nrf2 (ab89443), HO-1 (ab137749), SOD-2 (ab13533), T-NF-κB (ab7970), phosphor (P)-NF-κB (ab86299), T-Erk1/2 (ab36991), P-Erk1/2 (ab76299), T-IKKα/β (ab178870), P-IKKα/β (ab195907), β-actin (ab179467) and GAPDH (ab8245) (Abcam, Cambridge, MA, USA), cleaved caspase-9 (9505s), cleaved caspase-8 (8592s) (Cell Signaling, Danvers, MA, USA). After washing, the membranes were incubated with the secondary antibody at room temperature for 2 h and then detected with electrochemiluminescence detection kits (Merck Millipore, Billerica, MA, USA) for visualization. Protein bands were detected using ImageJ software (NIH, Bethesda, MD, USA).

### RT-PCR

RT-PCR was performed according to a method described previously with some modifications [75]. Briefly, the RNA was isolated from the spleens of BALB/c mice bearing with HepG2- and SMMC-7721-xenografted tumors using TRIzol (Invitrogen, USA) and then synthesized by QuantScript RT Kit (Tiangen Biotech (Beijing) Co. Ltd., China). β-actin primers were used as an internal control. The conditions of PCR amplification was shown as follows: denaturation at 95°C for 5 min, followed by 36 cycles at 95°C for 45 s, 57°C for 45 s, and 72°C for 45 s. The primer sequences are listed in Supplementary Table 2.

### Statistical analysis

The differences between the control and drug delivery groups were determined by a one-way analysis of variance (ANOVA). Post-hoc multiple comparisons (Dunn’s test) were performed using SPSS 16.0 software (IBM Corporation, Armonk, NY). A value of *P* < 0.05 was considered significant.

## Supplementary Material

Supplementary Figure 1

Supplementary Table 1

Supplementary Table 2
