# Supplementary material for: The anti-carcinogenesis properties of erianin in the modulation of oxidative stress-mediated apoptosis and immune response in liver cancer
Source: Aging (Albany NY). 2019 Nov 20;11(22):10284–300. doi: 10.18632/aging.102456 (PMC6914393; doi:10.18632/aging.102456)
Supplement: Supplementary Figure 1 [file aging-11-102456-s002..pdf]

SUPPLEMENTARY FIGURE

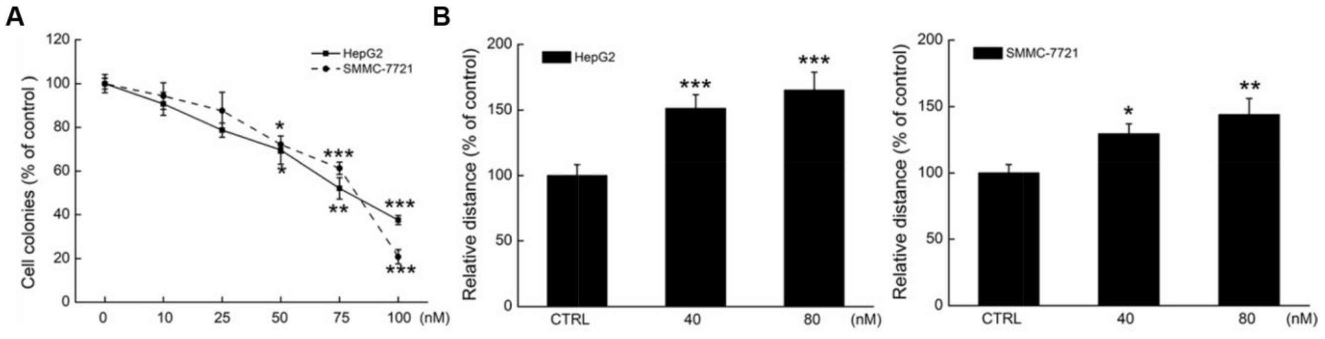

Supplementary Figure 1. (**A**) Erianin suppressed the formation of HepG2 and SMMC-7721 cell colonies. (**B**) Erianin inhibited the migration ability of HepG2 and SMMC-7721 cells after 24-h incubation analyzing via a wound healing assay. The distances of migrating cells were quantified using software Image J. Data are expressed as percentages relative to the corresponding control cells and as mean ± SD (n = 6). *P < 0.05, **P < 0.01, and ***P < 0.001 *vs* control cells.
