# Supplementary material for: The anti-carcinogenesis properties of erianin in the modulation of oxidative stress-mediated apoptosis and immune response in liver cancer
Source: Aging (Albany NY). 2019 Nov 20;11(22):10284–300. doi: 10.18632/aging.102456 (PMC6914393; doi:10.18632/aging.102456)
Supplement: Supplementary Table 1 [file aging-11-102456-s001..docx]

**Supplementary Table 1. The results of the Proteome Profler^TM^ Array using the Mouse XL Cytokine Kit**

| Coordinate& Target | HepG2- xenografted  tumor | | | SMMC-7721-xenografted tumor | | |
| --- | --- | --- | --- | --- | --- | --- |
|  | Pixel Density | | Fold | Pixel Density | | Fold |
|  | CTRL | Treated |  | CTRL | Treated |  |
| (A3-A4)(Adiponectin/Acrp30) | 0.8415 | 0.8636 | 1.03 | 0.7349 | 0.8408 | 1.14 |
| **(A5-A6)(Amphiregulin)** | **0.0793** | **0.2072** | **2.61** | 0.0984 | 0.1404 | 1.43 |
| **(A7-A8)(Angiopoietin-1)** | **0.0700** | **0.1457** | **2.08** | 0.0704 | 0.1048 | 1.49 |
| **(A9-A10)(Angiopoietin-2)** | **0.0778** | **0.1225** | **1.58** | **0.0866** | **0.1414** | **1.63** |
| (A11-A12)(Angiopoietin-like 3) | 0.1196 | 0.1439 | 1.20 | 0.1068 | 0.1298 | 1.22 |
| **(A13-A14)(BAFF/BLyS/TNFSF13B)** | **0.2080** | **0.1015** | **0.49** | 0.1159 | 0.1135 | 0.98 |
| (A15-A16)(C1q R1/CD93) | 0.1921 | 0.1751 | 0.91 | 0.1152 | 0.1334 | 1.16 |
| **(A17-A18)(CCL2/JE/MCP-1)** | 0.1226 | 0.1125 | 0.92 | **0.0562** | **0.0933** | **1.66** |
| **(A19-A20)(CCL3/CCL4 MIP-1 alpha/beta)** | 0.1190 | 0.1321 | 1.11 | **0.0784** | **0.1177** | **1.50** |
| (A21-A22)(CCL5/RANTES) | 0.3628 | 0.2438 | 0.67 | 0.2451 | 0.2002 | 0.82 |
| **(B3-B4)(CCL6/C10)** | **0.6013** | **0.9873** | **1.64** | 0.8929 | 0.9780 | 1.10 |
| **(B5-B6)(CCL11/Eotaxin)** | **0.1671** | **0.8583** | **5.14** | **0.4731** | **0.8059** | **1.70** |
| **(B7-B8)(CCL12/MCP-5)** | **0.1403** | **0.2995** | **2.13** | 0.1770 | 0.2058 | 1.16 |
| (B9-B10)(CCL17/TARC) | 0.1554 | 0.2321 | 1.49 | 0.2354 | 0.2196 | 0.93 |
| (B11-B12)(CCL19/MIP-3 beta) | 0.1823 | 0.1566 | 0.86 | 0.1155 | 0.1409 | 1.22 |
| (B13-B14)(CCL20/MIP-3 alpha) | 0.1094 | 0.1001 | 0.92 | 0.0682 | 0.0825 | 1.21 |
| (B15-B16)(CCL21/6Ckine) | 0.9453 | 0.6439 | 0.68 | 0.3447 | 0.4534 | 1.32 |
| (B17-B18)(CCL22/MDC) | 0.6063 | 0.3069 | 0.51 | 0.2763 | 0.2550 | 0.92 |
| **(B19-B20)(CD14)** | **0.1890** | **0.3878** | **2.05** | 0.4414 | 0.4328 | 0.98 |
| (B21-B22)(CD40/TNFRSF5) | 0.9843 | 0.9509 | 0.97 | 0.9106 | 0.9891 | 1.09 |
| **(C3-C4)(CD160)** | **0.1111** | **0.2244** | **2.02** | 0.1312 | 0.1709 | 1.30 |
| **(C5-C6)(Chemerin)** | **0.1572** | **0.3669** | **2.33** | 0.2721 | 0.3064 | 1.13 |
| **(C7-C8)(Chitinase 3-like 1)** | **0.4491** | **0.7344** | **1.64** | 0.7311 | 0.7302 | 1.00 |
| **(C9-C10)(Coagulation Factor III/ Tissue Factor)** | **0.5449** | **0.8791** | **1.61** | 0.9188 | 0.9378 | 1.02 |
| (C11-C12)(Complement Component C5/C5a) | 0.1135 | 0.1685 | 1.48 | 0.1634 | 0.1758 | 1.08 |
| (C13-C14)(Complement Factor D) | 0.3713 | 0.4959 | 1.34 | 0.3954 | 0.3355 | 0.85 |
| (C15-C16)(C-Reactive Protein/CRP) | 0.1291 | 0.0904 | 0.70 | 0.1132 | 0.0929 | 0.82 |
| **(C17-C18)(CX3CL1/Fractalkine)** | 0.1230 | 0.1063 | 0.86 | **0.0634** | **0.1061** | **1.67** |
| **(C19-C20)(CXCL1/KC)** | 0.0565 | 0.0774 | 1.37 | **0.0606** | **0.1075** | **1.77** |
| **(C21-C22)(CXCL2/MIP-2)** | 0.2261 | 0.2847 | 1.26 | **0.2602** | **0.6195** | **2.38** |
| (D1-D2)(CXCL9/MIG) | 0.2471 | 0.1599 | 0.65 | 0.1553 | 0.1365 | 0.88 |
| (D3-D4)(CXCL10/IP-10) | 0.0928 | 0.1246 | 1.34 | 0.0947 | 0.0894 | 0.94 |
| (D5-D6)(CXCL11/I-TAC) | 0.1092 | 0.1258 | 1.15 | 0.0990 | 0.1173 | 1.18 |
| **(D7-D8)(CXCL13/BLC/BCA-1)** | **0.6045** | **0.1780** | **0.29** | **0.5035** | **0.1555** | **0.31** |
| **(D9-D10)(CXCL16)** | **0.1359** | **0.2973** | **2.19** | 0.1955 | 0.2629 | 1.34 |
| (D11-D12)(Cystatin C) | 0.5241 | 0.5059 | 0.97 | 0.5072 | 0.4558 | 0.90 |
| (D13-D14)(DKK-1) | 0.1362 | 0.1295 | 0.95 | 0.1093 | 0.1258 | 1.15 |
| (D15-D16)(DPPIV/CD26) | 0.4565 | 0.4349 | 0.95 | 0.4889 | 0.4705 | 0.96 |
| (D17-D18)(EGF) | 0.1136 | 0.1371 | 1.21 | 0.1006 | 0.1225 | 1.22 |
| (D19-D20)(Endoglin/CD105) | 0.5968 | 0.7186 | 1.20 | 0.8732 | 0.7659 | 0.88 |
| (D21-D22)(Endostatin) | 0.3859 | 0.5752 | 1.49 | 0.4900 | 0.6139 | 1.25 |
| (D23-D24)(Fetuin A/AHSG) | 0.3725 | 0.3316 | 0.89 | 0.3175 | 0.3041 | 0.96 |
| (E1-E2)(FGF acidic) | 0.2768 | 0.3544 | 1.28 | 0.2715 | 0.1511 | 0.56 |
| **(E3-E4)(FGF-21)** | **0.0868** | **0.1310** | **1.51** | 0.1013 | 0.0914 | 0.90 |
| (E5-E6)(Flt-3 Ligand) | 0.1309 | 0.1636 | 1.25 | 0.1276 | 0.1321 | 1.04 |
| **(E7-E8)(Gas 6)** | **0.1604** | **0.2662** | **1.66** | 0.2337 | 0.2116 | 0.91 |
| (E9-E10)(G-CSF) | 0.1004 | 0.1376 | 1.37 | 0.1299 | 0.1270 | 0.98 |
| **(E11-E12)(GDF-15)** | **0.1339** | **0.2036** | **1.52** | 0.1779 | 0.1809 | 1.02 |
| (E13-E14)(GM-CSF) | 0.0535 | 0.0778 | 1.45 | 0.0565 | 0.0763 | 1.35 |
| **(E15-E16)(HGF)** | **0.1219** | **0.2009** | **1.65** | 0.2020 | 0.2169 | 1.07 |
| (E17-E18)(ICAM-1/CD54) | 0.2510 | 0.2443 | 0.97 | 0.1932 | 0.2491 | 1.29 |
| (E19-E20)(IFN-gamma) | 0.1194 | 0.1661 | 1.39 | 0.1207 | 0.1535 | 1.27 |
| **(E21-E22)(IGFBP-1)** | 0.0825 | 0.0971 | 1.18 | **0.0452** | **0.1092** | **2.42** |
| (E23-E24)(IGFBP-2) | 0.1203 | 0.1137 | 0.94 | 0.0783 | 0.1141 | 1.46 |
| **(F1-F2)(IGFBP-3)** | **0.1146** | **0.2065** | **1.80** | 0.2083 | 0.1894 | 0.91 |
| (F3-F4)(IGFBP-5) | 0.1778 | 0.1274 | 0.72 | 0.1015 | 0.0874 | 0.86 |
| (F5-F6)(IGFBP-6) | 0.1711 | 0.2244 | 1.31 | 0.2627 | 0.2036 | 0.78 |
| **(F7-F8)(IL-1 alpha/IL-1F1)** | **0.3661** | **0.6831** | **1.87** | 0.8255 | 0.7556 | 0.92 |
| **(F9-F10)(IL-1 beta/ IL-1F2)** | **0.0771** | **0.1316** | **1.71** | 0.1247 | 0.1431 | 1.15 |
| (F11-F12)(IL-1ra/IL-1F3) | 0.8026 | 0.9005 | 1.12 | 1.1160 | 0.9284 | 0.83 |
| **(F13-F14)(IL-2)** | **0.0291** | **0.0601** | **2.06** | **0.0405** | **0.0626** | **1.55** |
| **(F15-F16)(IL-3)** | **0.0282** | **0.0487** | **1.73** | **0.0225** | **0.0533** | **2.37** |
| (F17-F18)(IL-4) | 0.1535 | 0.1917 | 1.25 | 0.1325 | 0.1474 | 1.11 |
| (F19-F20)(IL-5) | 0.0990 | 0.1187 | 1.20 | 0.0803 | 0.0970 | 1.21 |
| **(F21-F22)(IL-6)** | **0.0328** | **0.0660** | **2.01** | **0.0283** | **0.0577** | **2.04** |
| (F23-F24)(IL-7) | 0.1339 | 0.1297 | 0.97 | 0.0876 | 0.1247 | 1.42 |
| (G1-G2)(IL-10) | 0.1391 | 0.1685 | 1.21 | 0.1286 | 0.1437 | 1.12 |
| (G3-G4)(IL-11) | 0.1357 | 0.1790 | 1.32 | 0.1482 | 0.1405 | 0.95 |
| **(G5-G6)(IL-12p40)** | **0.1072** | **0.1709** | **1.59** | 0.1466 | 0.1549 | 1.06 |
| **(G7-G8)(IL-13)** | **0.1117** | **0.1830** | **1.64** | **0.1475** | **0.2425** | **1.64** |
| (G9-G10)(IL-15) | 0.1751 | 0.2062 | 1.18 | 0.1945 | 0.2102 | 1.08 |
| **(G11-G12)(IL-17A)** | **0.0636** | **0.1964** | **3.09** | **0.1190** | **0.1877** | **1.58** |
| **(G13-G14)(IL-22)** | **0.0680** | **0.1098** | **1.61** | 0.0960 | 0.0981 | 1.02 |
| (G15-G16)(IL-23) | 0.0893 | 0.1032 | 1.16 | 0.0848 | 0.0896 | 1.06 |
| (G17-G18)(IL-27p28) | 0.1193 | 0.1284 | 1.08 | 0.0981 | 0.1048 | 1.07 |
| (G19-G20)(IL-28) | 0.1848 | 0.1928 | 1.04 | 0.1397 | 0.1416 | 1.01 |
| **(G21-G22)(IL-33)** | **0.3786** | **0.7798** | **2.06** | 0.7374 | 0.6642 | 0.90 |
| (G23-G24)(LDL R) | 0.1321 | 0.1476 | 1.12 | 0.0990 | 0.1412 | 1.43 |
| (H1-H2)(Leptin) | 0.1350 | 0.1328 | 0.98 | 0.1069 | 0.1287 | 1.20 |
| (H3-H4)(LIF) | 0.1582 | 0.1899 | 1.20 | 0.1518 | 0.1691 | 1.11 |
| (H5-H6)(Lipocalin-2/NGAL) | 0.1682 | 0.2179 | 1.30 | 0.2115 | 0.2235 | 1.06 |
| **(H7-H8)(LIX)** | **0.1984** | **0.7776** | **3.92** | 0.9024 | 0.9474 | 1.05 |
| (H9-H10)(M-CSF) | 0.1326 | 0.1955 | 1.47 | 0.1963 | 0.2058 | 1.05 |
| **(H11-H12)(MMP-2)** | **0.2205** | **0.4858** | **2.20** | 0.5599 | 0.3918 | 0.70 |
| **(H13-H14)(MMP-3)** | **0.1471** | **0.9295** | **6.32** | 0.9977 | 0.9079 | 0.91 |
| **(H15-H16)(MMP-9)** | **0.0289** | **0.1221** | **4.22** | 0.1356 | 0.0965 | 0.71 |
| (H17-H18)(Myeloperoxidase) | 0.5474 | 0.5428 | 0.99 | 0.4504 | 0.4495 | 1.00 |
| **(H19-H20)(Osteopontin (OPN))** | **0.2032** | **0.5258** | **2.59** | 0.2896 | 0.3231 | 1.12 |
| (H21-H22)(Osteoprotegerin/ TNFRSF11B) | 0.0589 | 0.0802 | 1.36 | 0.0650 | 0.0656 | 1.01 |
| (H23-H24)(PD-ECGF/ Thymidine phosphorylase) | 0.0577 | 0.0725 | 1.26 | 0.0567 | 0.0541 | 0.95 |
| (I1-I2)(PDGF-BB) | 0.1994 | 0.1889 | 0.95 | 0.1679 | 0.1637 | 0.98 |
| (I3-I4)(Pentraxin 2/SAP) | 0.1090 | 0.1300 | 1.19 | 0.1055 | 0.1083 | 1.03 |
| (I5-I6)(Pentraxin 3/ TSG-14) | 0.1655 | 0.1860 | 1.12 | 0.2805 | 0.1780 | 0.63 |
| **(I7-I8)(Periostin/OSF-2)** | **0.0694** | **0.2605** | **3.76** | 0.2920 | 0.2161 | 0.74 |
| (I9-I10)(Pref-1/DLK-1/FA1) | 0.1526 | 0.2248 | 1.47 | 0.2380 | 0.1796 | 0.75 |
| (I11-I12)(Proliferin) | 0.1244 | 0.1856 | 1.49 | 0.1611 | 0.1553 | 0.96 |
| **(I13-I14)(Proprotein Convertase 9/ PCSK9)** | **0.1235** | **0.2277** | **1.84** | 0.2032 | 0.2677 | 1.32 |
| (I15-I16)(RAGE) | 0.1268 | 0.1433 | 1.13 | 0.1218 | 0.1132 | 0.93 |
| (I17-I18)(RBP4) | 0.3691 | 0.3731 | 1.01 | 0.2634 | 0.3000 | 1.14 |
| (I19-I20)(Reg3G) | 0.5676 | 0.6992 | 1.23 | 0.6470 | 0.7520 | 1.16 |
| (I21-I22)(Resistin) | 0.7348 | 0.6490 | 0.88 | 0.5178 | 0.2880 | 0.56 |
| (J3-J4)(E-Selectin/CD62E) | 0.0779 | 0.0749 | 0.96 | 0.0651 | 0.0633 | 0.97 |
| (J5-J6)(P-Selectin/CD62P) | 0.1202 | 0.1681 | 1.40 | 0.2043 | 0.1543 | 0.75 |
| **(J7-J8)(Serpin E1/PAI-1)** | **0.1358** | **0.2465** | **1.82** | 0.3715 | 0.3040 | 0.82 |
| (J9-J10)(Serpin F1/PEDF) | 0.7509 | 0.9841 | 1.31 | 1.0655 | 0.9161 | 0.86 |
| (J11-J12)(Thrombopoietin) | 0.0949 | 0.1313 | 1.38 | 0.1229 | 0.1071 | 0.87 |
| (J13-J14)(TIM-1/KIM-1/ HAVCR) | 0.2240 | 0.1506 | 0.67 | 0.1291 | 0.1404 | 1.09 |
| (J15-J16)(TNF-alpha) | 0.0844 | 0.1019 | 1.21 | 0.0840 | 0.0892 | 1.06 |
| (J17-J18)(VCAM-1/CD106) | 0.3437 | 0.3171 | 0.92 | 0.2483 | 0.2227 | 0.90 |
| (J19-J20)(VEGF) | 0.1312 | 0.1780 | 1.36 | 0.1330 | 0.1574 | 1.18 |
| **(J21-J22)(WISP-1/CCN4)** | **0.1241** | **0.4796** | **3.86** | **0.7897** | **0.3270** | **0.41** |

The marked word indicates that the change is > 50%.
