# Supplementary material for: The anti-carcinogenesis properties of erianin in the modulation of oxidative stress-mediated apoptosis and immune response in liver cancer
Source: Aging (Albany NY). 2019 Nov 20;11(22):10284–300. doi: 10.18632/aging.102456 (PMC6914393; doi:10.18632/aging.102456)
Supplement: Supplementary Table 2 [file aging-11-102456-s003..pdf]

SUPPLEMENTARY TABLES

Please browse Full Text version to see the data of Supplementary Table 1

Supplementary Table 1. The results of the Proteome Profler™ Array using the Mouse XL Cytokine Kit.

Supplementary Table 2. The primer sequences used in RT-PCR.

Fragment size (bp)	Name	Sequence (5′ to 3′)
342	HO-1-F	GCCCTGGAAGAGGAGATAGA
	HO-1-R	GTCGATGTTCGGGAAGGTAAA
288	SOD-1-F	CGGATGAAGAGAGGCATGTT
	SOD-1-R	GGTTTGAGGGTAGCAGATGAG
137	β-actin-F	ATCGTGCGAGACATCAATG
	β-actin-R	TCGTTGCCTATTGTGATGAC
